# Relevance of Simultaneous Mono-Ubiquitinations of Multiple Units of PCNA Homo-Trimers in DNA Damage Tolerance

**DOI:** 10.1371/journal.pone.0118775

**Published:** 2015-02-18

**Authors:** Rie Kanao, Yuji Masuda, Saori Deguchi, Mayumi Yumoto-Sugimoto, Fumio Hanaoka, Chikahide Masutani

**Affiliations:** 1 Department of Genome Dynamics, Research Institute of Environmental Medicine, Nagoya University, Nagoya, Japan; 2 Graduate School of Frontier Biosciences, Osaka University, Osaka, Japan; 3 Department of Toxicogenomics, Nagoya University Graduate School of Medicine, Nagoya, Japan; 4 Faculty of Science, Gakushuin University, Tokyo, Japan; University of Hawaii Cancer Center, UNITED STATES

## Abstract

DNA damage tolerance (DDT) pathways, including translesion synthesis (TLS) and additional unknown mechanisms, enable recovery from replication arrest at DNA lesions. DDT pathways are regulated by post-translational modifications of proliferating cell nuclear antigen (PCNA) at its K164 residue. In particular, mono-ubiquitination by the ubiquitin ligase RAD18 is crucial for Polη-mediated TLS. Although the importance of modifications of PCNA to DDT pathways is well known, the relevance of its homo-trimer form, in which three K164 residues are present in a single ring, remains to be elucidated. Here, we show that multiple units of a PCNA homo-trimer are simultaneously mono-ubiquitinated *in vitro* and *in vivo*. RAD18 catalyzed sequential mono-ubiquitinations of multiple units of a PCNA homo-trimer in a reconstituted system. Exogenous PCNA formed hetero-trimers with endogenous PCNA in WI38VA13 cell transformants. When K164R-mutated PCNA was expressed in these cells at levels that depleted endogenous PCNA homo-trimers, multiple modifications of PCNA complexes were reduced and the cells showed defects in DDT after UV irradiation. Notably, ectopic expression of mutant PCNA increased the UV sensitivities of Polη-proficient, Polη-deficient, and REV1-depleted cells, suggesting the disruption of a DDT pathway distinct from the Polη- and REV1-mediated pathways. These results suggest that simultaneous modifications of multiple units of a PCNA homo-trimer are required for a certain DDT pathway in human cells.

## Introduction

The stability of genomic DNA is challenged by various DNA-damaging agents. Replication fork progression is disrupted by DNA lesions; therefore, cells have evolved DNA damage tolerance (DDT) pathways that rescue stalled replication forks. Translesion synthesis (TLS) and template switch (TS) are the two major mechanisms of DDT. TLS is catalyzed by specialized DNA polymerases that incorporate nucleotides opposite DNA lesions [1]. Human DNA polymerase η (Polη), the product of the xeroderma pigmentosum variant (XP-V) gene that is responsible for inherited predisposition to cancer [2, 3], plays a prominent role in DDT of UV-induced lesions. The precise mechanism of TS is still under debate, although it is thought to be a homology-dependent system that uses newly synthesized sister strands to bypass lesions [4].

DDT pathways are controlled by post-translational modifications of proliferating cell nuclear antigen (PCNA) [5]. PCNA forms a ring-shaped homo-trimer complex that interacts with many proteins and plays crucial roles in DNA replication, DNA repair, and cell-cycle control [6]. In response to replication blockage, PCNA is mono-ubiquitinated at its K164 residue by the RAD18 ubiquitin (Ub) ligase [7]. Members of the Y-family of DNA polymerases, including Polη, have Ub-binding motifs [8] and interact preferentially with mono-ubiquitinated PCNA [1], which enables switching from replicative DNA polymerases at stalled replication sites. In addition, it has been reported that Ub-PCNA promotes not only Polη-dependent TLS but also a Polη-independent DDT pathway [9, 10], and also multiple two TLS polymerases mechanisms have been proposed [11, 12]. However, mechanisms to promote these multiple TLS pathways still need to be elucidated. Furthermore, current models propose that K63-linked poly-ubiquitination at K164 of PCNA promotes TS [4], which is catalyzed by the E3 Ub ligases Rad5 (yeast) or HLTF and SHPRH (mammals) [13]. PCNA is also modified by SUMO at K164 [7]; this modification prevents undesirable recombination during DNA replication and regulates TS [4, 14].

One PCNA ring consists of three PCNA monomers, meaning that three K164 residues are present in a single ring. Although the importance of post-translational modifications of PCNA to the control of DDT pathways is well known, the relevance of its homo-trimer form has not been examined in detail. Here, we show that multiple units of a PCNA ring undergo simultaneous mono-ubiquitination both *in vitro* and *in vivo*. Experiments using human cells ectopically expressing wild-type (WT) or K164-mutated PCNA revealed that mutation of this lysine residue disrupts mono-ubiquitination of the multiple units within a homo-trimer complex as well as a DDT pathway that is distinct from Polη-mediated TLS.

## Materials and Methods

### Generation of DNA constructs

The human *PCNA* cDNA sequence was cloned into the pET21 vector (Clontech) to produce pET/PCNA-WT. The pET/PCNA[KR] construct was generated by mutation of lysine 164 (K164) in *PCNA* to arginine (R) using the MutanK kit (Takara) and an oligomer (5′-CACTCCGTCTCGTGCACAGG-3′). The silent mutations at the PCNA-specific siRNA target sequences were produced using the AMAP Mutagenesis Kit (MBL) and the following primers: 5′-CTCTATTGTGACGGCCTCTTCCTCTTTATC-3′ and 5′-CCTTCTTCGTCTTCTATTTTCGGAGCCAAG-3′. The resulting siRNA-resistant PCNA sequences were subcloned into the pMK10 expression vector to produce pMK10/PCNA-WT(resistant) and pMK10/PCNA[KR](resistant). HA-tagged PCNA was produced by inserting oligonucleotides encoding the HA-tag into the pET constructs, resulting in the formation of pET/HA-PCNA-WT and pET/HA-PCNA[KR]. The HA-tagged PCNA fragments were then subcloned into the pIRESneo2 or pIREShyg3 vector (Invitrogen). Silent mutations at the siRNA target sequences were introduced into the pIREShyg3 constructs. The expression construct for FLAG-tagged Polη was produced by inserting synthesized FLAG oligomers (5′-CTAGCCATATGGACTACAAAGACGATGACGACAAGG-3′ and 5′-AATTCCTTGTCGTCATCGTCTTTGTAGTCCATATGG-3′) into a pIRESneo2/Polη construct (15). The GFP-tagged Polη construct, pAcGFP/Polη, was produced by inserting the Polη cDNA sequence into the pAcGFP1-Hyg-C1 vector (Clontech). The FLAG-RAD18 expression vector was prepared as described previously [16]. The Ub fragment was obtained from a pCAGGS/HA-Ub construct (a gift from Dr. K. Sugasawa at Kobe University, Japan) and subcloned into the pET28a vector to produce His-Ub. The His-Ub fragment was then subcloned into pIREShyg3 to generate pIREShyg3/His-Ub. To prepare the FLAG/HA/His-PCNA constructs, the PCNA open reading frame sequence was subcloned into the pET28 vector to generate pET28/His-PCNA-WT. The [KR] mutation (K164R) was introduced as described above. To prepare the pET28/His-PCNA[KR]-Ub construct, the PCNA[KR](resistant) fragment was obtained by PCR using primers (5′-CGACTGCTTAAGATTTCGAGGCGCGCCTGGTCCAG-3′ and 5′-CCTATCGCTAGCTCCAGCTCCACCCGCAGATCCTTCTTCATCC-3′) that were designed to eliminate the stop codon. The fragment was subcloned into the pIREShyg3 vector along with the Ub fragment derived from pCAGGS/HA-Ub. A stop codon was introduced at glycine 74 of the Ub sequence using the QuikChange II XL Site-Directed Mutagenesis Kit (Stratagene) and the following primers: 5′-CTGCGCTTGAGGTAGGGTGTCTAAG-3′ and 5′-CTTAGACACCCTACCTCAAGCGCAG-3′. The PCNA[KR]-Ub fragment was then subcloned into the pET28 vector to generate pET28/His-PCNA[KR]-Ub. An *Nco*I site located in the open reading frame of the sequence encoding PCNA in the pET28/His-PCNA-WT, pET28/His-PCNA[KR] and pET28/His-PCNA[KR]-Ub constructs was disrupted using the QuikChange Lightning Mutagenesis Kit (Stratagene) and the following oligomers: 5′-GACCGCAACCTGGCAATGGGCGTGAACCTC-3′ and 5′-GAGGTTCACGCCCATTGCCAGGTTGCGGTC-3′. FLAG- or HA-tagged PCNA sequences were produced by replacing the His-tag sequences located between the *Nco*I and *Nde*I sites in the pET28 constructs with synthesized FLAG (5′-CATGGACTACAAAGACGATGACGACAAGCTCGTGCCGCGCGGCAGCCA-3′ and 5′-TATGGCTGCCGCGCGGCACGAGCTTGTCGTCATCGTCTTTGTAGTC-3′) or HA (5′-CATGGGCTACCCATACGATGTTCCGGATTACGCTAGTCTCGTGCCGCGCGGCAGCCA-3′ and 5′-TATGGCTGCCGCGCGGCACGAGACTAGCGTAATCCGGAA CATCGTATGGGTAGCC-3′) DNA oligomers, respectively. The tagged PCNA-WT and PCNA[KR] sequences were obtained by *Xba*I/*Xho*I digestions of these constructs, followed by blunt-ending using the Klenow enzyme. The tagged PCNA[KR]-Ub sequences were obtained by *Xba*I/*Not*I digestions and blunt-ending was performed using Klenow. To generate the pET28/FLAG-PCNA-WT/HA-PCNA[KR]/His-PCNA[KR] construct, the His-PCNA[KR] fragment was subcloned into the blunt-ended *Xho*I site of the pET28/HA-PCNA[KR] construct, and then the FLAG-PCNA-WT fragment was subcloned into the blunt-ended *Xba*I site of the pET28/HA-PCNA[KR]/His-PCNA[KR] construct. To generate the pET28/FLAG-PCNA-WT/HA-PCNA[KR]-Ub/His-PCNA[KR] and pET28/FLAG-PCNA-WT/HA-PCNA[KR]-Ub/His-PCNA[KR]-Ub constructs, the FLAG-PCNA-WT fragment was subcloned into the blunt-ended *Xho*I site of the pET28/HA- PCNA[KR]-Ub construct, and then the His-PCNA[KR] and His-PCNA[KR]-Ub fragments were subcloned into the blunt-ended *Not*I site of the pET28/FLAG-PCNA-WT/HA- PCNA[KR]-Ub construct.

### siRNAs

The PCNA-specific siRNA (5′-UAUGGUAACAGCUUCCUCCdTdT-3′), REV1-specific siRNA [15], and non-targeting control siRNA were purchased from Dharmacon.

### Antibodies

The following antibodies were used: PCNA (FL261 and PC10; Santa Cruz Biotechnology), HA (3F10; Roche), Polη [15], REV1 [15], LaminB (C20; Santa Cruz Biotechnology), RAD18 (rabbit polyclonal), phospho-CHK1 (Ser345) (133D3; Cell Signaling), CHK1 (G4; Santa Cruz Biotechnology), actin (I19; Santacruz), γH2AX (Millipore), Ub (P4D1; Santa Cruz Biotechnology), and FLAG (M2; Sigma).

### Preparation of Flag-, HA-, and His-tagged PCNA proteins

The following procedures were performed at 4°C using the FPLC and SMART systems (GE Healthcare). Soluble materials from *Escherichia coli* BL21 (DE3) cells were dissolved in buffer A (20 mM sodium phosphate (pH 7.2), 0.3 M NaCl, 10% glycerol, and 10 mM β-mercaptoethanol), passed through Hitrap DEAE (GE Healthcare), and then loaded onto TALON resin (Clontech). After sequential washes with buffer A and buffer B (20 mM Tris-HCl (pH 8.0), 0.1 M NaCl, 10% glycerol, and 10 mM β-mercaptoethanol), the bound materials were eluted with 0.2 M imidazole in buffer B and loaded onto anti-FLAG M2 agarose. After a wash with buffer B, the bound materials were eluted with buffer B containing 0.1 mg/ml FLAG peptide (Sigma) and then loaded onto anti-HA-agarose (Sigma). After a further wash with buffer B, the bound materials were eluted with buffer B containing 0.1 mg/ml HA peptide (Sigma). The PCNA-enriched fractions detected by SDS-PAGE and Coomassie Brilliant Blue staining were loaded onto a MonoQ/PC1.6/5 column (GE Healthcare) and the proteins were eluted with a linear gradient of NaCl (0.1–0.5 M) in 20 mM sodium phosphate (pH 7.2), 0.1 mM EDTA, 10% glycerol, and 10 mM β-mercaptoethanol.

### *In vitro* PCNA ubiquitination assay

The PCNA ubiquitination assay was performed as described previously [16], with minor modifications. In brief, reaction mixtures containing 20 mM HEPES-NaOH (pH 7.5), 50 mM NaCl, 0.2 mg/ml bovine serum albumin, 1 mM *dithiothreitol*, 10 mM MgCl_2_, 1 mM ATP, 100 ng poly(dA)-oligo(dT), 40 nM PCNA, 14 nM RFC, 34 nM E1, 6.8 μM Ub, and 22 nM RAD6A-RAD18 were incubated at 30°C for the indicated times.

### Transfection

Transfections were performed using the NEON electroporation transfection system (Invitrogen) or FuGENE6 reagent (Roche).

### Generation of stable transformants

Human WI38VA13 cells (established fibroblasts [17]) were transfected with empty pMK10 vector or with the pMK10/PCNA-WT(resistant) or PCNA[KR](resistant) construct. Clones were selected using 0.2 mg/ml G418. XP2SASV3 cells (kindly provided by Dr. Kiyoji Tanaka, Osaka University, Japan) were transfected with empty pIRES vector or with the pIREShyg3/HA-PCNA-WT(resistant) or HA-PCNA[KR](resistant) construct, and clones were selected using 0.2 mg/ml hygromycin. WI38VA13 cells were transfected with the pIRESneo2/HA-PCNA-WT and pIRESneo2/HA-PCNA[KR], and clones were then selected using 0.2 mg/ml G418. WI38VA13/His-Ub cells were obtained by selection with 0.1 mg/ml hygromycin after transfection of cells with the pIREShyg3/His-Ub construct.

### His-Ub pull-down assay

Cells were lysed in lysis buffer comprising 20 mM Tris-HCl (pH 7.5), 0.15 M KCl, 0.2 mM EDTA, 25% glycerol, 0.5% NP-40, 1.5 mM MgCl_2_, 1× Complete Protease Inhibitor Cocktail (Roche), and 1× Phosphatase Inhibitor Cocktail Set II (Calbiochem). After removal of detergent-soluble materials by centrifugation, the precipitants were resuspended in microccocal nuclease buffer comprising 20 mM Tris-HCl (pH 7.5), 0.1 M KCl, 0.3 M sucrose, 0.1% Triton X-100, 2 mM MgCl_2_, 1 mM CaCl_2_, 1× EDTA-free Complete protease inhibitor cocktail (Roche), and 2.5 U of micrococcal nuclease (Roche). The mixtures were incubated at room temperature for 10 min and then centrifuged at 300 *g* for 5 min. The solubilized (chromatin) fractions were mixed with Ni-NTA agarose (Qiagen) at 4°C in binding buffer (20 mM sodium phosphate (pH 7.2), 10% glycerol, 0.1% Triton X-100, 0.25 mM phenylmethylsulfonyl fluoride, and 20 mM imidazole) containing 0.5 M NaCl. After three washes with binding buffer containing 1 M NaCl, the bound proteins were eluted with binding buffer containing 0.5 M NaCl and 250 mM imidazole.

### Immunoprecipitation assays

Chromatin fractions were prepared from cells expressing HA-PCNA-WT or HA-PCNA[KR] as described above and then incubated with anti-HA agarose (Sigma) at 4°C for 3 h. After washing the beads with wash buffer (20 mM Tris-HCl (pH 7.5), 100 mM KCl, 10% glycerol, 5 mM MgCl_2_, 0.1% Tween-20, 0.2 mM EDTA, 0.2 mM phenylmethylsulfonyl fluoride, and 0.2 mM β-mercaptoethanol), the precipitated proteins were eluted by incubation with wash buffer containing 0.5 mg/ml HA peptide (Sigma).

### Clonogenic cell survival

Cells were seeded into 10 cm dishes at a density of 2,000–3,000 cells per dish, incubated overnight, irradiated with UVC or treated with cisplatin (Nichi-Iko Pharmaceutical) for 24 h, and then allowed to form colonies. The siRNA treatments were performed 3 days prior to seeding.

### Cell synchronization

Cells were incubated with medium containing 2.5 mM thymidine for 24 h, medium without thymidine for 14 h, and then medium containing 2.5 mM thymidine for a further 24 h. The cells were then washed with phosphate-buffered saline (PBS), irradiated or mock-irradiated with UV, washed twice with medium lacking thymidine, and cultured in medium without thymidine. Cells were transfected with the FLAG-Polη and FLAG-RAD18 constructs 36 h prior to synchronization.

### FACS analysis

Cells were harvested and fixed with 70% ethanol at -20°C. The fixed cells were treated with 0.1 mg/ml RNase A, stained with 50 μg/ml propidium iodide (Nacalai Tesque), and then analyzed using a FC500 flow cytometer (Beckman). Data were analyzed using FlowJo software (TOMY Digital Biology).

### Detection of CHK1 phosphorylation

Cells were irradiated with 10 J/m^2^ UVC and incubated for the indicated periods. Proteins were extracted with NP-40 buffer comprising 20 mM Tris-HCl (pH 7.5), 150 mM NaCl, 10% glycerol, 1 mM *dithiothreitol*, 0.5% NP-40, 0.25 mM phenylmethylsulfonyl fluoride, 0.2 μg/ml antipain, 0.2 μg/ml aprotinin, 0.1 μg/ml leupeptin, 0.08 μg/ml pepstatin, 0.05 mM EGTA, and 1× Phosphatase Inhibitor Cocktail Set II (Calbiochem), and then the soluble fractions were analyzed by immunoblotting using anti-phospho-CHK1 (S345), anti-CHK1, or anti-actin antibodies.

### Detection of γH2AX positive cells

Cells were fixed with 4% paraformaldehyde in PBS for 10 min and then permeabilized with 1% SDS and 0.5% Triton X-100 in PBS for 10 min at room temperature. The cells were incubated with anti-γH2AX antibodies for 30 min at 37°C. Alexa Fluor 594-conjugated secondary antibodies (Invitrogen) were used. Nuclei were stained with 1 μg/ml Hoechst 33342 and images were collected using a LSM710 confocal microscope (Zeiss).

### Detection of GFP-Polη foci

WI38VA13/PCNA-WT and WI38VA13/PCNA[KR] cells were transfected with nontargeting or PCNA-specific siRNA. After incubation for 3 days, GFP-Polη was transiently introduced into these cells. Thirty-six hours after transfection, cells were irradiated with UVC at 15 J/m^2^ and then incubated for 3 h at 37°C. Triton-soluble materials were removed by incubating the cells with 0.5% Triton X-100 in PBS supplemented with 0.4 μg/ml antipain, 0.4 μg/ml aprotinin, 0.2 μg/ml leupeptin, 0.16 μg/ml pepstatin, 0.1 mM EGTA, and 0.5 mM phenylmethylsulfonyl fluoride. Cells were fixed with 3.5% formaldehyde in PBS and then permeabilized by treatment with 0.1% Triton X-100 and 3.5% formaldehyde in PBS. After sequential treatments with 70% EtOH, 100% EtOH, and acetone on ice, cells were incubated with an anti-PCNA antibody. Alexa Fluor 594-conjugated secondary antibodies (Invitrogen) were used to visualize the immune-conjugated proteins and images were collected using a LSM710 confocal microscope (Zeiss).

## Results

### RAD6-RAD18 preferentially catalyzes multiple mono-ubiquitinations of PCNA homo-trimers *in vitro*

**Fig 1:**
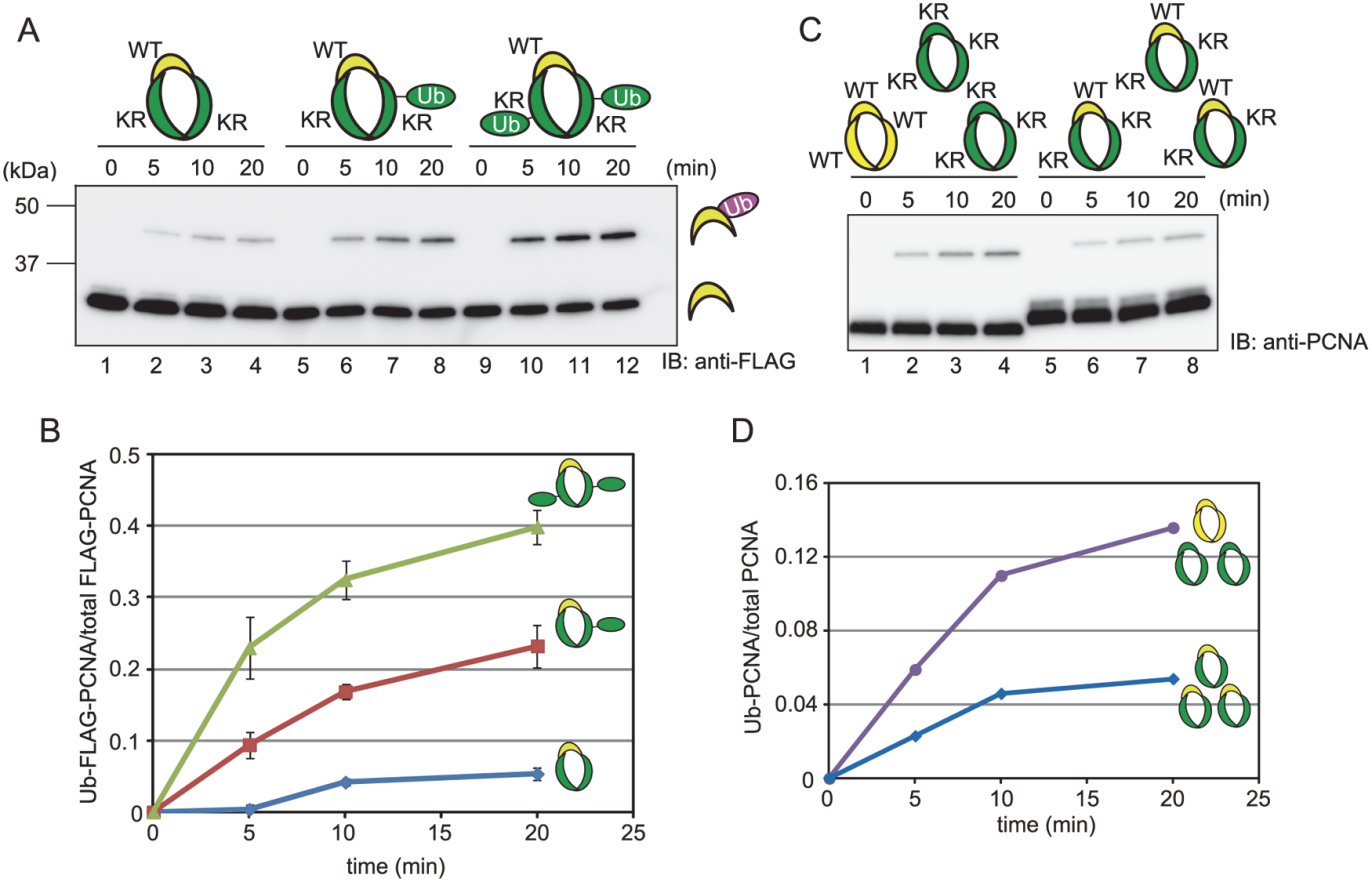
PCNA homo-trimers undergo multiple-mono-ubiquitinations *in vitro*. (A, B) The results of an *in vitro* mono-ubiquitination assay of triple-tagged PCNA hetero-trimers performed using purified RAD6-RAD18. The yellow and green shapes indicate wild-type (WT) and K164R (KR) PCNA molecules, respectively. (A) Immunoblot analysis using an anti-FLAG antibody of PCNA complexes containing a WT monomer that was or was not ubiquitinated. (B) The ratios of ubiquitinated to total FLAG-PCNA at the indicated time points. Data are represented as the mean ± standard deviation (SD) of n = 3 independent experiments. (C, D) The results of an *in vitro* mono-ubiquitination assay of a PCNA hetero-trimer consisting of one modifiable (WT) unit and two unmodifiable (K164R) units, and a 1:2 ratio of WT and K164R PCNA homo-trimers. (C) Immunoblot analysis using an anti-PCNA antibody. (D) The ratios of ubiquitinated to total PCNA at the indicated time points.

To determine whether multiple units in a PCNA homo-trimer can be ubiquitinated simultaneously and whether the number of ubiquitinated units of a PCNA homo-trimer influence the rate of subsequent ubiquitination of modifiable units, the substrate preference of the RAD6-RAD18 ubiquitination complex was examined using an *in vitro* assay. The following three recombinant PCNA complexes were generated and used in the assay: i) a hetero-trimer comprising a unit of FLAG-tagged modifiable WT PCNA (PCNA-WT), a unit of HA-tagged K164R mutated PCNA (PCNA[KR]), and a unit of His-tagged PCNA[KR]; ii) a hetero-trimer comprising a unit of FLAG-tagged PCNA-WT, a unit of HA-tagged Ub-fused PCNA[KR] (PCNA[KR]-Ub), and a unit of His-tagged PCNA[KR]; and iii) a hetero-trimer comprising a unit of FLAG-tagged PCNA-WT, a unit of HA-tagged PCNA[KR]-Ub, and a unit of His-tagged PCNA[KR]-Ub (Fig. 1A). These complexes were purified using three affinity resins (FLAG, HA, and His) and were therefore expected to comprise all three components of the trimer. Mono-ubiquitination of these PCNA hetero-trimers was proportional to the number of Ub fusion monomers in the complex (Fig. 1A and B). RAD6-RAD18 catalyzed mono-ubiquitination preferentially in the complex containing two PCNA[KR]-Ub fusion units, followed by one PCNA[KR]-Ub fusion unit, and then zero PCNA[KR]-Ub fusion units, suggesting that RAD18 preferentially promotes sequential mono-ubiquitinations of PCNA trimers.

Next, we performed a titration experiment and found that mono-ubiquitination of PCNA-WT homo-trimers was reduced by the addition of PCNA[KR] homo-trimers in a proportion-dependent manner (S1 Fig. A and B). Specifically, in a 1:2 mixture of PCNA-WT homo-trimers to PCNA[KR] homo-trimers, the level of mono-ubiquitination of the WT molecules was approximately one-third of that in a solution of PCNA-WT homo-trimers alone (S1B Fig.). It was reported previously that RAD18 predominantly mono-ubiquitinates PCNA molecules loaded onto DNA, and that the K164R mutation does not affect this process or the ability of PCNA to act as a clamp for DNA polymerase δ [18]. Therefore, it is likely that the PCNA[KR] homo-trimers competed with the PCNA-WT homo-trimers for loading onto DNA, thereby impeding mono-ubiquitination of the WT molecules by RAD18. In parallel, we examined the level of mono-ubiquitination of PCNA hetero-trimers comprising one WT and two mutant molecules. In this experiment, despite the maintenance of the same ratio of WT to mutant monomers used in the previous experiment, mono-ubiquitination of the WT PCNA molecules in the hetero-trimers was lower than that of the WT molecules in a 1:2 mixture of WT homo-trimers and PCNA[KR] homo-trimers (Fig. 1C and D, and S1 Fig, A and B). This result is consistent with those shown in Fig. 1A, which demonstrate that RAD18 preferentially catalyzes sequential mono-ubiquitinations of the second and third units of a trimer rather than one-shot mono-ubiquitination of the first unit.

### PCNA homo-trimers undergo multiple mono-ubiquitinations in human cells

**Fig 2:**
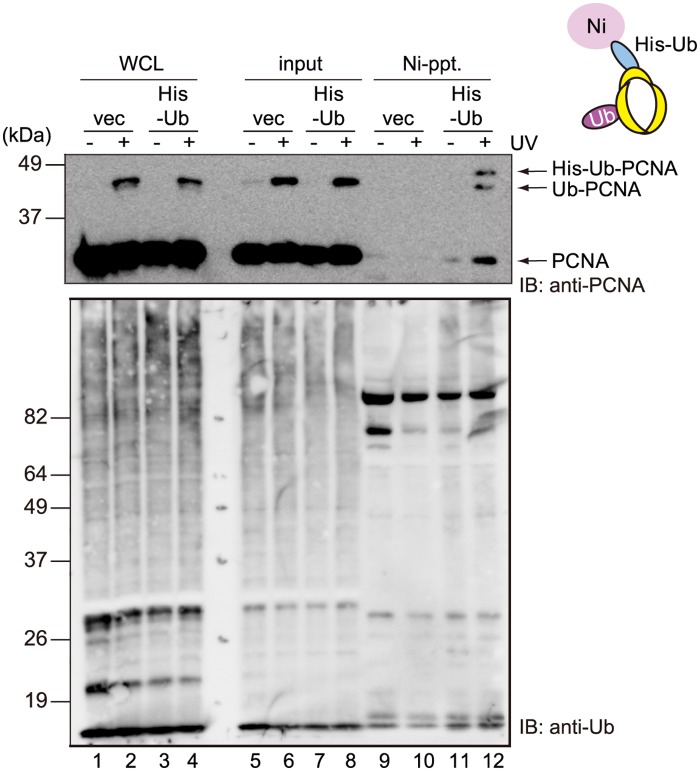
PCNA homo-trimers undergo multiple-mono-ubiquitinations *in vivo*. The results of a pull-down assay using stably-expressed His-Ub. WI38VA13 cells stably expressing His-Ub (His-Ub) or vector-transfected control (vec) were mock-irradiated (-) or irradiated with 15 J/m^2^ UVC (+) and incubated for 3 h. Ni-pull-down assays were performed using the chromatin fractions (2% input) and samples were analyzed by immunoblotting using an anti-PCNA or anti-Ub antibody. WCL, whole cell lysate.

Next, we investigated whether multiple units of the PCNA trimer are modified simultaneously in human cells. For this purpose, human WI38VA13 fibroblasts stably expressing His-tagged Ub together with endogenous Ub were generated. These cells were mock-irradiated or irradiated with 15 J/m^2^ UVC and incubated for 3 h, and then analyzed by immunoblotting using an anti-Ub or anti-PCNA antibody. The Ub signal intensity of the extract from WI38VA13 cells stably expressing His-Ub was comparable to that of the extract from vector-transfected cells (Fig. 2, compare lanes 1–2 with 3–4), indicating that the expression level of His-Ub was much lower than that of endogenous Ub. Consequently, PCNA molecules containing His-Ub modifications were rarely detected in the chromatin fractions, even after UV irradiation (Fig. 2, lane 8). However, Ni-pull-down followed by immunoblotting with an anti-PCNA antibody revealed that larger molecules with a slower migration speed were recovered along with unmodified molecules and those containing endogenous Ub modifications (Ub-PCNA) (Fig. 2, lane 12). The larger protein identified by Ni-affinity precipitation showed a similar migration to that of the PCNA molecule modified with His-Ub by RAD6-RAD18 *in vitro* [16]; therefore, we assumed that it represented PCNA mono-ubiquitinated with His-Ub. Like recombinant PCNA trimers, the co-precipitated complexes were resistant to a high salt (1 M NaCl) wash (S2 Fig.), suggesting that the precipitants had formed stable trimers. These results suggest that a portion of PCNA trimers are simultaneously mono-ubiquitinated on multiple subunits after UV irradiation in human cells.

### Ectopically expressed mutant PCNA disturbs multiple mono-ubiquitinations of PCNA trimers

**Fig 3:**
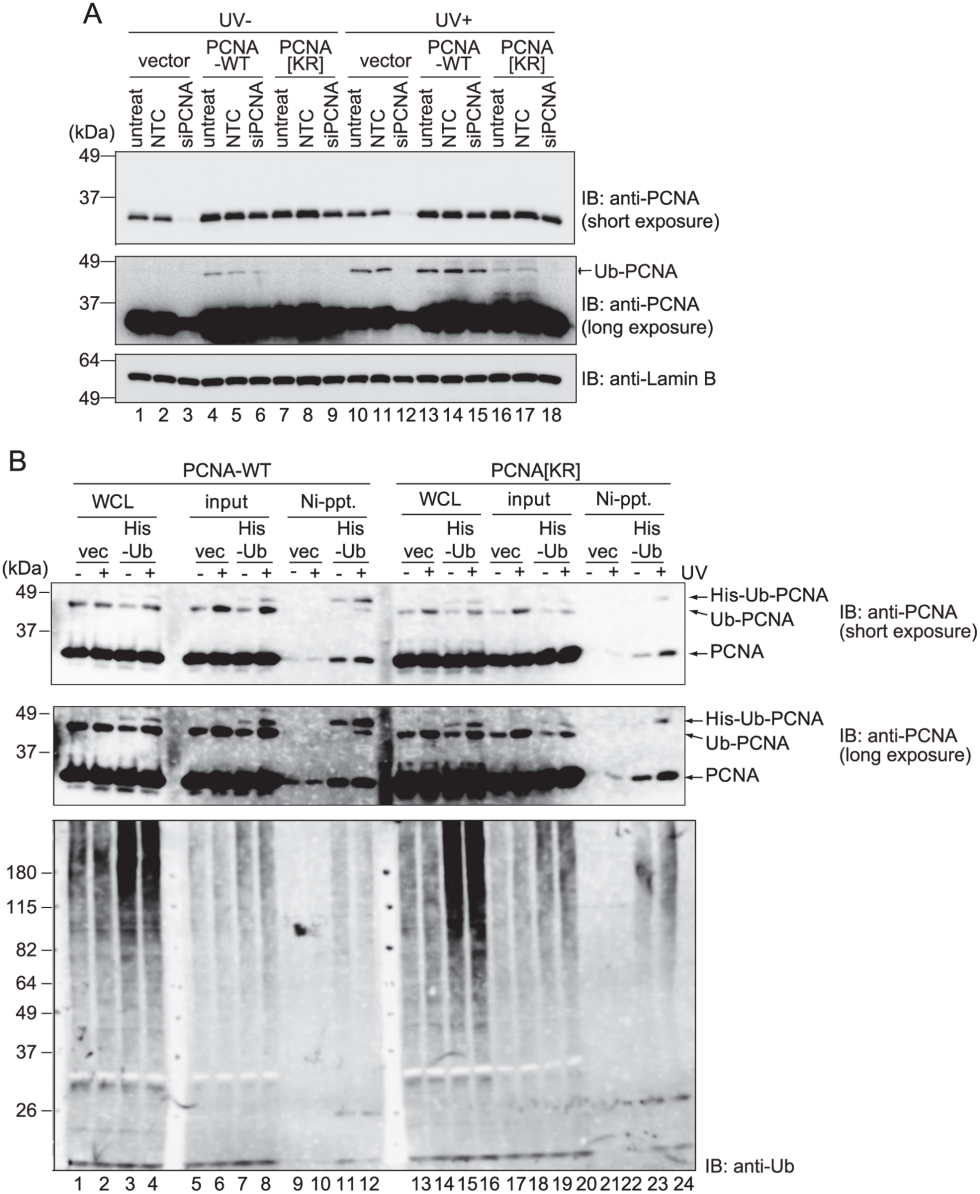
Ectopic expression of K164R-mutated PCNA disturbs multiple mono-ubiquitinations of PCNA trimers in human cells. (A) Immunoblot analyses of endogenous and ectopically expressed PCNA in vector-transfected, PCNA-wild-type (WT)-expressing and PCNA[KR]-expressing WI38VA13 cells that were untreated (untreat) or transfected with a nontargeting control (NTC) or PCNA-specific (siPCNA) siRNA. Four days after transfection, the cells were mock-irradiated (UV-) or irradiated with UVC at 10 J/m^2^ (UV+), and then incubated for a further 3 h. Lamin B was detected as a loading control. (B) Ni-pull-down assay of PCNA-WT and PCNA[KR] cells transiently transfected with empty vector (vec) or the His-Ub expression construct (His-Ub). The cells were incubated for 36 h, mock-irradiated (-) or irradiated with 15 J/m^2^ UVC (+), and then incubated for a further 3 h. Ni-pull-down assays were performed using the chromatin fractions (5% input) and samples were analyzed by immunoblotting using an anti-PCNA or anti-Ub antibody. WCL, whole cell lysate.

To examine the physiological relevance of the multiple-mono-ubiquitination of PCNA, we attempted to disturb this process by expressing unmodifiable PCNA molecules in human cells. WI38VA13 cells stably expressing exogenous PCNA-WT or PCNA[KR] together with endogenous PCNA were generated. The PCNA expression constructs contained mutations that rendered them resistant to degradation by a PCNA-specific siRNA, which was confirmed experimentally (Fig. 3A, lanes 6, 9, 15, and 18; see also S3 Fig.). Exogenous PCNA[KR] was not ubiquitinated after suppression of endogenous PCNA expression by this siRNA (Fig. 3A, lane 18). To determine whether multiple mono-ubiquitination of PCNA trimers is suppressed in cells expressing exogenous PCNA[KR], His-Ub was transiently introduced into these cells and those expressing exogenous PCNA-WT. Immunoblotting using an anti-Ub antibody revealed that the Ub signal intensities in whole cell lysates were increased markedly following expression of exogenous His-Ub (Fig. 3B, compare lanes 1–2 with lanes 3–4, and lanes 13–14 with lanes 15–16), indicating that His-Ub was expressed at higher levels in the transient transfectants than the stable transformants. Hence, after UV irradiation, His-Ub-modified PCNA molecules were observed in the chromatin fractions of cells expressing PCNA-WT or PCNA[KR] (Fig. 3B, lanes 8 and 20). However, whereas a Ni-pull-down experiment recovered Ub-PCNA together with His-Ub-PCNA-WT, Ub-PCNA did not co-precipitate with His-Ub-PCNA[KR] (Fig. 3B, compare lane 12 of short exposure with lane 24 of long exposure). This result suggests that simultaneous ubiquitination of multiple units of PCNA trimers was lower in the cells expressing PCNA[KR] than those expressing PCNA-WT.

### A certain DNA damage tolerance pathway is disrupted by ectopic expression of mutant PCNA

**Fig 4:**
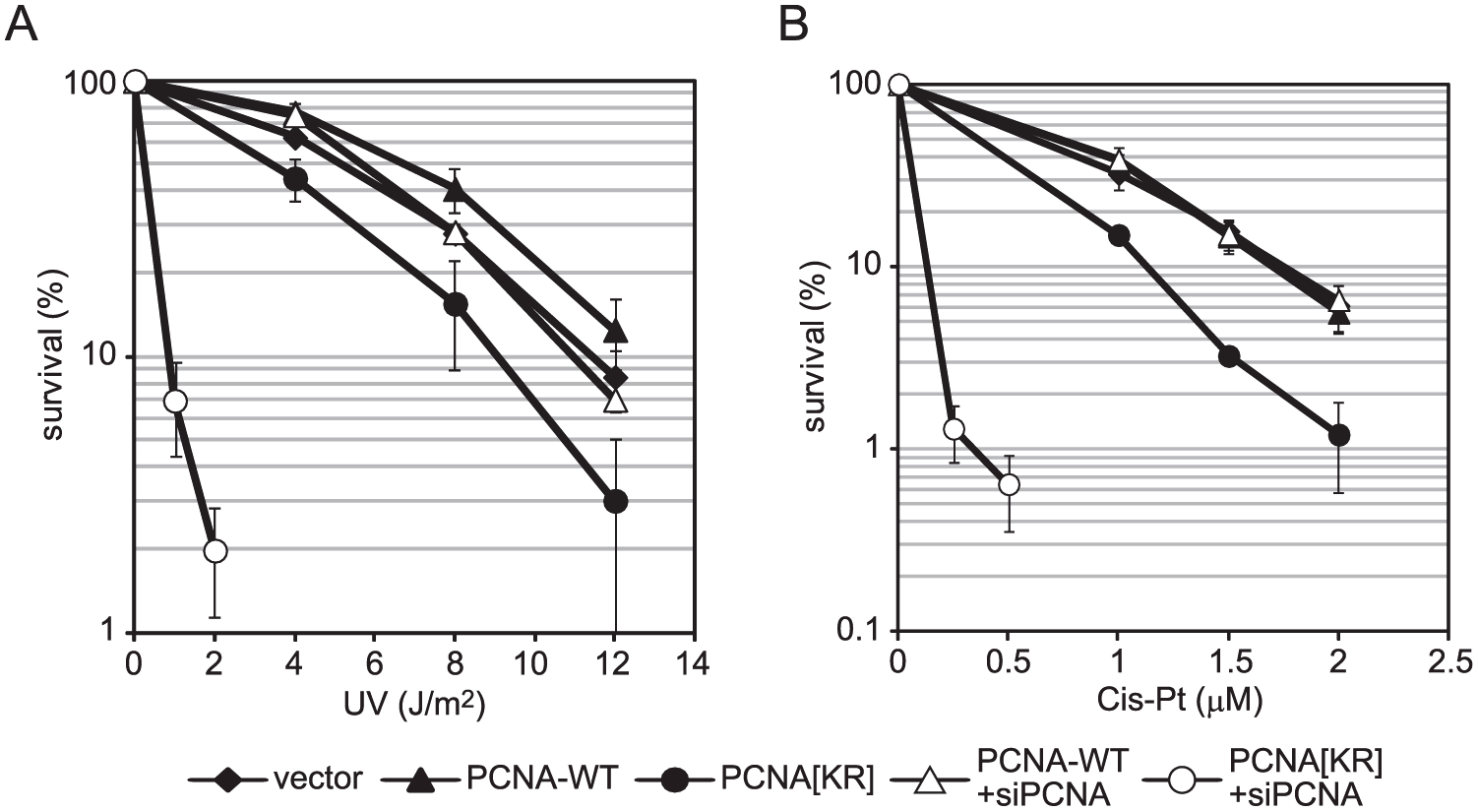
PCNA[KR] cells are sensitive to DNA-damaging agents. The sensitivities of WI38VA13-derived cells to UV irradiation (A) or cisplatin (Cis-Pt) (B) with and without siRNA-mediated knockdown of endogenous PCNA (siPCNA). Clonogenic survival rates were determined after UVC irradiation or cisplatin treatment (24 h) of the indicated cell strains. Data are represented as the mean ± standard deviation of n = 3 independent experiments.

**Fig 5:**
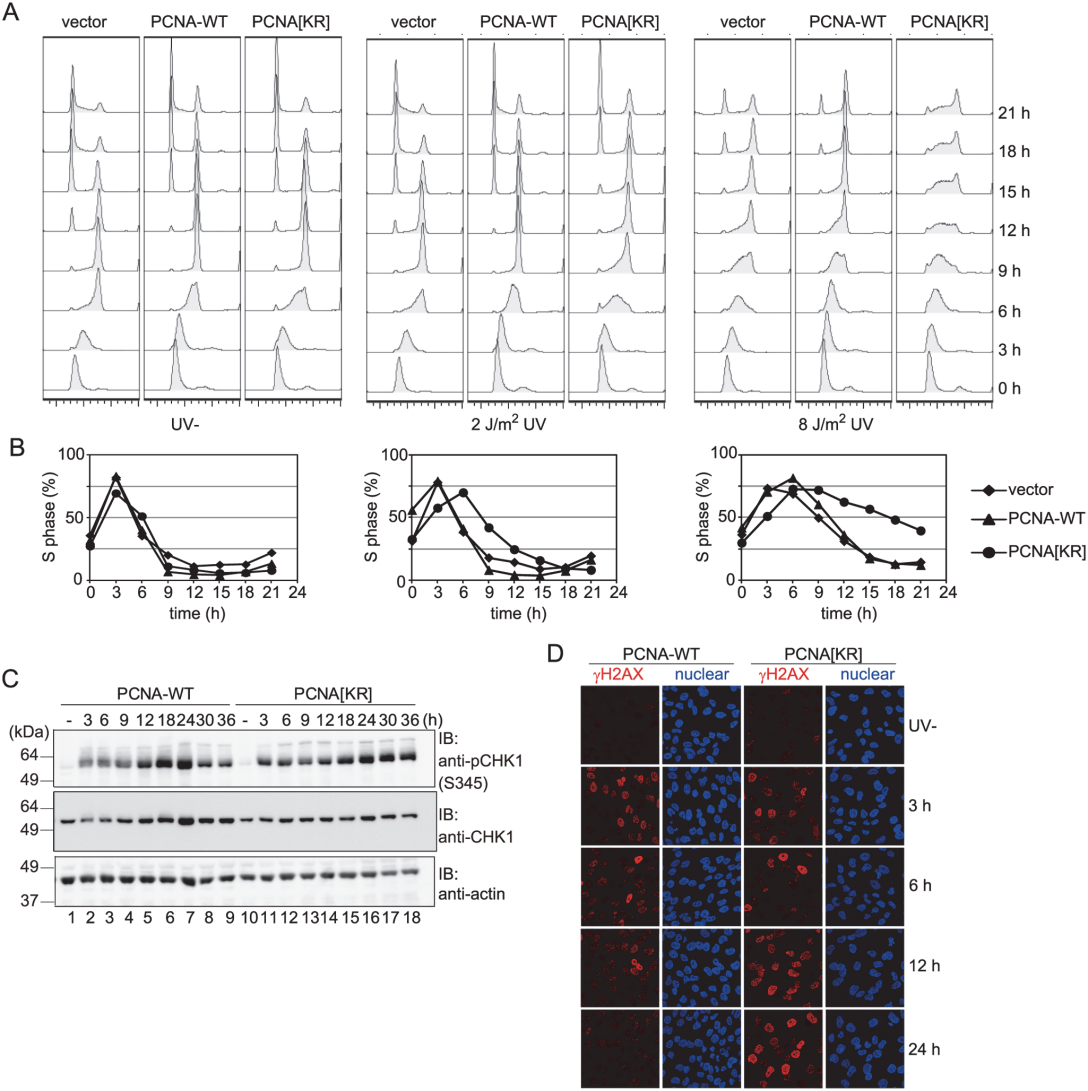
PCNA[KR] cells show DNA replication problems after UV irradiation. (A, B) S-phase progression analysis. Vector-transfected, PCNA-wild-type (WT) or PCNA[KR] WI38VA13 cells were synchronized using the double thymidine block method, mock-irradiated (UV-) or irradiated with UVC at the released time point, and then incubated for the indicated periods prior to FACS analysis of the cell-cycle profile. (C) Immunoblot analysis of CHK1 phosphorylation. Cells were irradiated with 10 J/m^2^ UVC and incubated for the indicated periods. Immunoblotting was performed using anti-phospho-CHK1 (Ser345), anti-CHK1 and anti-actin antibodies. (D) Analysis of γH2AX-positive populations. The indicated cells were irradiated with 5 J/m^2^ UVC and incubated for the indicated periods. The cells were then fixed and stained with an anti-γH2AX antibody (shown in red). Nuclei were visualized by Hoechst 33342 staining (shown in blue).

siRNA-mediated suppression of endogenous PCNA resulted in marked increases in the sensitivities of PCNA[KR] cells to UV (Fig. 4A) and cisplatin (Fig. 4B), indicating that modifications at K164 of PCNA are required for proper functioning of DDT pathways in human cells. Notably, even prior to the siRNA treatment, PCNA[KR] cells were slightly but reproducibly more sensitive to UV and cisplatin than PCNA-WT cells (Fig. 4A and B), suggesting that the DDT pathway is partially disturbed in PCNA[KR] cells. To investigate the effect of DNA damage on replication of PCNA[KR] cells, S-phase progression was examined. Vector-transfected, PCNA-WT and PCNA[KR] cells were synchronized by the double thymidine block method, irradiated with UV at the released time, and then incubated for up to 21 h. S-phase delay was observed in PCNA[KR] cells exposed to 2 J/m^2^ or 8 J/m^2^ of UV irradiation; this effect was especially evident at the higher dose (Fig. 5 A and B). This result suggests that a certain DDT pathway is disturbed in PCNA[KR] cells.

Phosphorylation of the S345 residue of CHK1 in response to DNA damage is required for the DNA damage checkpoint response. Immunoblot analyses of cells irradiated with UV and then incubated for up to 36 h showed that UV-induced phosphorylation of S345 of CHK1 was more persistent in PCNA[KR] than PCNA-WT cells (Fig. 5C). Furthermore, γH2AX-positive populations, which are indicative of DNA breaks, were also observed for longer periods in irradiated PCNA[KR] cells than irradiated PCNA-WT cells (Fig. 5D). Taken together, these results suggest that replication problems are prolonged in PCNA[KR] cells.

### Ectopically expressed mutant PCNA forms hetero-trimers with endogenous monomers, depletes endogenous homo-trimers, and disturbs the DDT pathway

**Fig 6:**
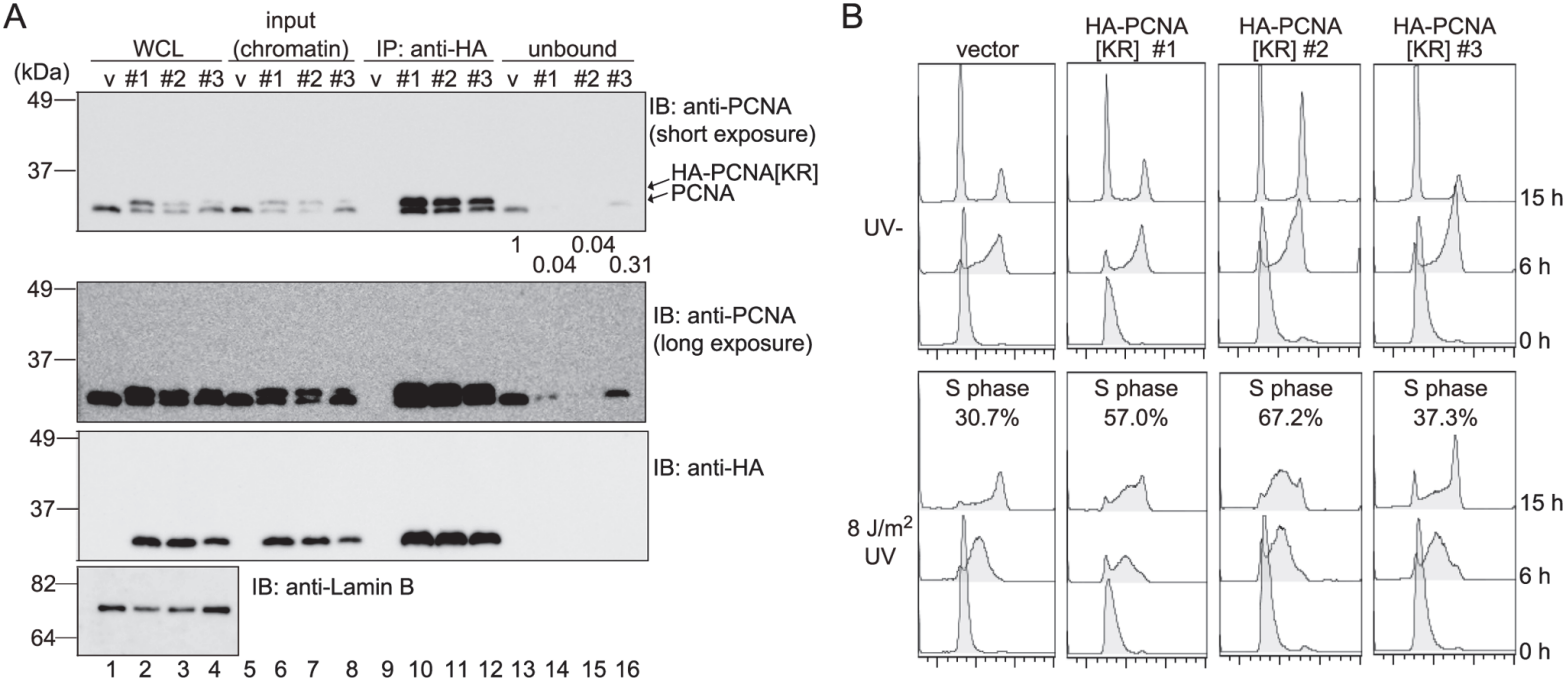
Ectopic K164R-mutated PCNA forms hetero-trimers with endogenous PCNA, depletes endogenous PCNA homo-trimers, and disturbs DDT. (A) Immunoprecipitation assays showing the formation of hetero-trimers of exogenous PCNA[KR] and endogenous PCNA in WI38VA13 cell transformants. The chromatin fractions (20% input) prepared from vector-transfected cells (v) and cells expressing varying levels of HA-PCNA[KR] (clone #1 = #2 > #3) were immunoprecipitated using an anti-HA antibody and then analyzed by immunoblotting using an anti-PCNA, anti-HA, or anti-Lamin B (control) antibody. The whole cell lysates (WCLs) and 20% unbound samples were also analyzed. The ratios of the signal intensities of endogenous PCNA detected in the unbound fractions of clones #1, #2, and #3 to control cells (v) are indicated below the upper panel. (B) Cell-cycle progression analyses of vector-transfected WI38VA13 cells and the HA-PCNA[KR] clone #1, #2 and #3 cells. The cells were synchronized by the double thymidine block method, mock-irradiated (UV-) or irradiated with 8 J/m^2^ UVC (UV+) at the released time point, and then incubated for the indicated periods. The cell-cycle profiles were obtained by FACS analysis. The percentages of cells in the S-phase at the 15 h time point are indicated.

To confirm that the inhibitory effect of the K164R mutation on simultaneous ubiquitination of multiple units of PCNA trimers was due to the formation of hetero-complexes of exogenous PCNA[KR] monomers and endogenous PCNA monomers, WI38VA13 cells stably expressing HA-tagged PCNA[KR] at different levels (clone #1 = clone #2 > clone #3) were established. For clones #1 and #2, the ectopic HA-PCNA[KR] molecules were present in the chromatin fractions at levels similar to that of endogenous PCNA (Fig. 6A, lanes 6 and 7), and for clone #3, the ectopic PCNA molecules were also detected, albeit at a lower level than that of endogenous PCNA (Fig. 6A, lane 8). Both endogenous PCNA and HA-PCNA[KR] were co-precipitated from the chromatin fractions of cells from all three clones by an anti-HA antibody (Fig. 6A, lanes 10–12). Gel filtration analysis also confirmed that these two types of PCNA formed well-blended hetero-trimers (S2 Fig.). Very small amounts of endogenous PCNA were detected in the unbound fractions of cells from clones #1 and #2 (Fig. 6A, lanes 14 and 15), indicating that endogenous PCNA homo-trimers were effectively depleted from the cells expressing relatively high levels of HA-PCNA[KR]. By contrast, approximately 30% of the endogenous PCNA in vector-transfected cells was detected in the unbound fraction of cells from clone #3 (Fig. 6A, lane 16), indicating that the cells expressing lower levels of HA-PCNA[KR] still expressed some triple modification-susceptible endogenous PCNA homo-trimers. Notably, S-phase delay after UV irradiation was evident in HA-PCNA[KR] cells from clones #1 and #2, from which multiple modification-susceptible PCNA trimers were effectively depleted, but S-phase delay was mild in cells from clone #3, which retained some endogenous PCNA homo-trimers (Fig. 6B). These results suggest that disruption of the DDT pathway in PCNA[KR] cells may be caused by depletion of multiple modification-susceptible PCNA homo-trimers.

We reckoned that ectopic PCNA expression in the untagged PCNA[KR] cells was higher than endogenous PCNA expression because the level of siRNA-resistant PCNA in the untagged PCNA[KR] cells was approximately 1.7-fold higher than the level of endogenous PCNA in the vector-transfected cells (Fig. 3A, compare lanes 1 and 9). Taken together, these findings suggest that the number of endogenous PCNA homo-trimers capable of undergoing triple modifications was substantially lower in the cells expressing PCNA[KR] than the vector-transfected cells or those expressing PCNA-WT.

### Forced increase of the PCNA mono-ubiquitination level does not restore the S-phase delay of cells expressing mutant PCNA

**Fig 7:**
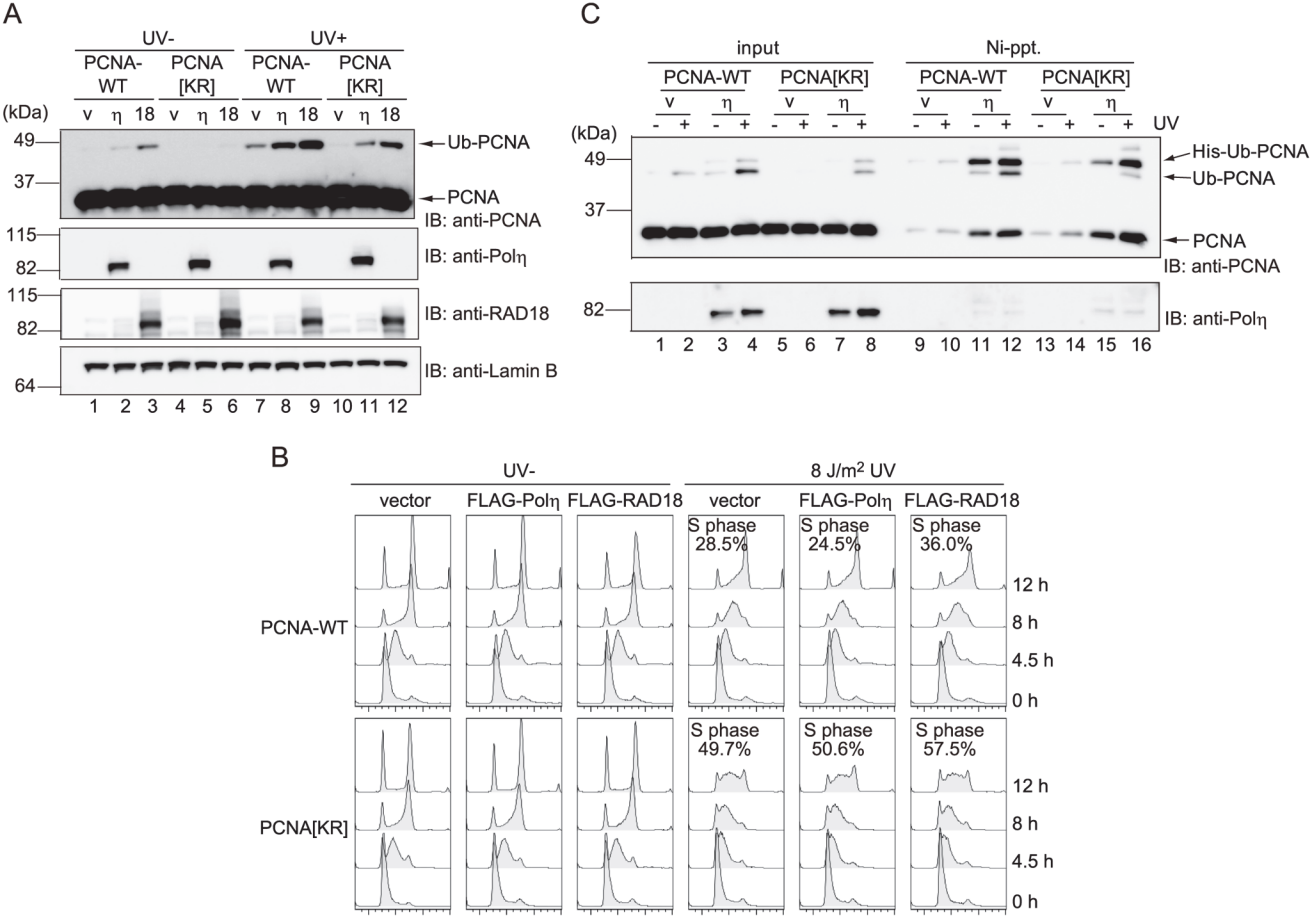
Forced increase of mono-ubiquitinated PCNA does not restore the DDT defects of PCNA[KR] cells. PCNA-wild-type (WT) and PCNA[KR] cells were transfected with empty vector (v) or FLAG-Polη (η) or FLAG-RAD18 (18) expression constructs. (A) Cells were mock-irradiated (UV-) or irradiated with 15 J/m^2^ UVC (UV+), incubated for 3 h, and then subjected to immunoblotting using an anti-PCNA, anti-Polη, anti-RAD18, and anti-Lamin B antibodies. (B) Cells were co-transfected with GFP and empty vector or the FLAG-Polη or FLAG-RAD18 expression construct, and then treated with 2.5 mM thymidine for 24 h to concentrate the G1/S-phase populations. The cells were then mock-irradiated (UV-) or irradiated with 8 J/m^2^ UV and cultured for the indicated periods. The cell-cycle profiles of the GFP-positive populations were determined by FACS analysis. The percentages of cells in the S-phase populations at the 12 h time point are indicated. (C) Cells were co-transfected with His-Ub and empty vector (v) or the FLAG-Polη (η) expression construct, mock-irradiated (-) or irradiated with 15 J/m^2^ UVC (+), and then incubated for 3 h. Ni-pull-down assays were performed using the chromatin fractions (10% input) and samples were analyzed by immunoblotting using an anti-PCNA or anti-Polη antibody.

To determine whether the quantity or quality of PCNA mono-ubiquitination is important for directing the DDT pathway, the levels of mono-ubiquitinated endogenous and ectopically expressed PCNA were examined in PCNA-WT and PCNA[KR] cells. The level of mono-ubiquitinated PCNA in both cell types was increased by ectopic overexpression of RAD18 [19] or Polη [20] and was enhanced markedly after UV irradiation (Fig. 7A, compare lanes 1–6 with lanes 7–12). Following UV irradiation, the level of mono-ubiquitinated PCNA in PCNA[KR] cells overexpressing Polη (Fig. 7A, lane 11) was similar to that in PCNA-WT vector control cells (Fig. 7A, lane 7). Similarly, the level of mono-ubiquitinated PCNA in PCNA[KR] cells overexpressing RAD18 (Fig. 7A, lane 12) was higher than in the PCNA-WT vector control cells. However, a FACS analysis of cells co-transfected with GFP and empty vector or the FLAG-Polη or FLAG-RAD18 expression construct revealed that the delayed S-phase progression of UV-irradiated PCNA[KR] cells was not restored by the increased PCNA mono-ubiquitination level caused by overexpression of RAD18 or Polη (Fig. 7B). Pull-down experiments with transiently expressed His-Ub confirmed that smaller amounts of Ub-PCNA were recovered with His-Ub-PCNA from PCNA[KR] cells expressing ectopic Polη (Fig. 7C, lane 16) than with His-Ub-PCNA from the equivalent PCNA-WT cells (Fig. 7C, lane 12). Formation of hetero-trimers of exogenous HA-PCNA[KR] and endogenous PCNA on chromatin was not affected by ectopic overexpression of Polη or RAD18 (data not shown). These results suggest that multiple mono-ubiquitinations rather than increased levels of bulk mono-ubiquitination are crucial for the DDT pathway. Slight but detectable amounts of multiple mono-ubiquitinated PCNA complexes were observed in PCNA[KR] cells overexpressing Polη (Fig. 7C); nevertheless, S-phase delay was not restored in these cells, indicating that increased multiple ubiquitinations, which presumably consist of modifications on two PCNA units, are insufficient to direct the DDT pathway. This finding is consistent with the observation that the cells lacking endogenous triple modification-susceptible PCNA homo-trimers showed DDT defects (Fig. 6).

### The DDT pathway disturbed by ectopic expression of mutant PCNA is distinct from Polη- and Rev1-mediated TLS

**Fig 8:**
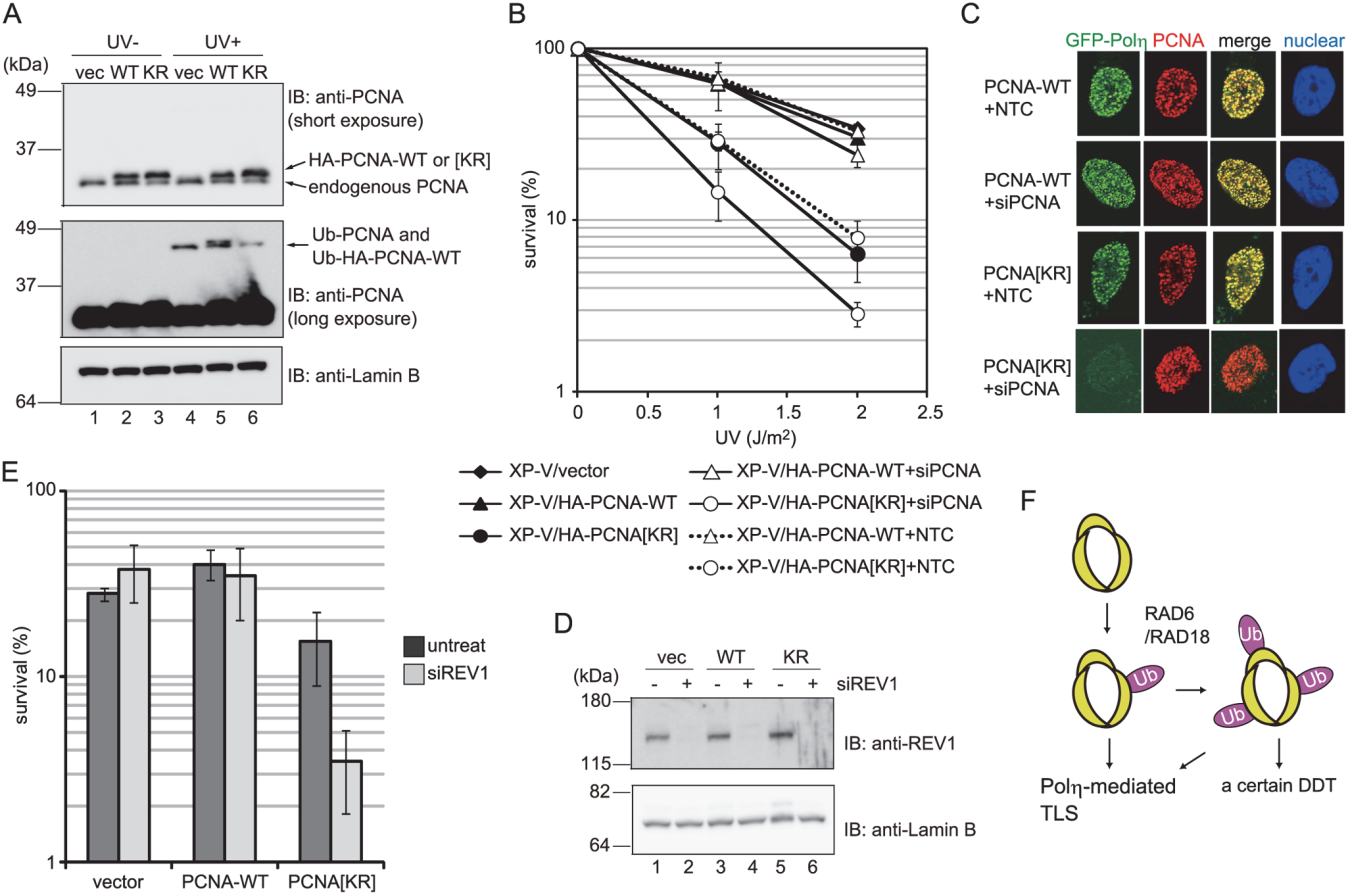
The DDT pathway disturbed by expression of PCNA[KR] is distinct from Polη- and Rev1-mediated TLS. (A) Immunoblot analyses of PCNA expression in Polη-deficient XP2SASV3 cells stably expressing empty vector (vec), HA-PCNA-wild-type (WT) or HA-PCNA[KR] (KR). The cells were mock-irradiated (UV-) or irradiated with UVC at 10 J/m^2^ (UV+) and then incubated for 3 h. Whole cell lysates were analyzed by immunoblotting using an anti-PCNA or anti-Lamin B (control) antibody. (B) The UVC sensitivities of Polη-deficient XP2SASV3-derived cells (XP-V) expressing empty vector, HA-PCNA-WT or HA-PCNA[KR] after treatment with an siRNA to knockdown endogenous PCNA (siPCNA) or a nontargeting control siRNA (NTC). Data are represented as the mean ± standard deviation (SD) of n = 3 independent experiments. (C) GFP-Polη was transiently expressed in WI38VA13/PCNA-WT or WI38VA13/PCNA[KR] cells treated with an siPCNA or a NTC. The cells were irradiated with 15 J/m^2^ UVC and then incubated for 3 h. After extraction of Triton-soluble materials and fixation, PCNA was detected using an anti-PCNA antibody and nuclei were visualized by Hoechst 33342 staining. A typical nucleus of each cell type is shown. (D) Immunoblot analyses of REV1 and Lamin B (control) expression in WI38VA13 cells expressing empty vector (vec), PCNA-WT (WT) or PCNA[KR] (KR), and transfected with or without a REV1-specific siRNA. Four days after transfection, whole cell lysates were analyzed by immunoblotting using an anti-REV1 or anti-Lamin B antibody. (E) The effects of knockdown of REV1 on the UV sensitivities of the cells described in (D). The ratios of surviving cells exposed to 8 J/m^2^ of UVC irradiation to those that were mock-irradiated were determined. The survival rates of the cells that were not treated with the REV1-specific siRNA (untreat) were taken from Fig. 4A. Data are represented as the mean ± SD of n = 3 independent experiments. (F) Schematic illustration of PCNA mono-ubiquitination-mediated DDT pathways in human cells.

Polη-mediated TLS is a prominent DDT pathway induced by UV lesions. To examine the nature of the specific DDT pathway disturbed by expression of PCNA[KR], Polη-deficient XP-V (XP2SASV3) cells stably expressing HA-tagged PCNA-WT or PCNA[KR] were prepared. Compared with the XP-V/HA-PCNA-WT and vector control cells, the XP-V/HA-PCNA[KR] cells, in which HA-PCNA[KR] expression was higher than that of endogenous PCNA (Fig. 8A), displayed increased sensitivity to UV irradiation (Fig. 8B). This result suggests that the DDT pathway disrupted by PCNA[KR] expression is independent of Polη-mediated TLS. The sensitivity of the XP-V/HA-PCNA[KR] cells to UV was increased only slightly by siRNA-mediated suppression of endogenous PCNA (Fig. 8B), suggesting that PCNA[KR] expression alone was sufficient to abolish the DDT pathway in Polη-deficient cells. Furthermore, UV-induced formation of transiently expressed GFP-Polη foci in WI38VA13 cells expressing PCNA[KR] was abolished by treatment with a specific siRNA to knockdown endogenous PCNA, but not by treatment with a nontargeting control siRNA (Fig. 8C). By contrast, UV-induced formation of GFP-Polη foci in WI38VA13 cells expressing PCNA-WT was not affected by knockdown of endogenous PCNA. These results suggest that expression of the K164R mutant of PCNA does not affect the Polη-mediated TLS pathway but does disrupt an alternative DDT pathway, and that the Polη-mediated TLS pathway is inactivated following suppression of endogenous PCNA.

The dCMP transferase REV1 promotes TLS of UV lesions in cooperation with Polζ [1]; therefore, the effect of siRNA-mediated knockdown of REV1 on the UV sensitivities of WI38VA13/PCNA[KR] and WI38VA13/PCNA-WT cells was examined (Fig. 8D). Knockdown of REV1 did not alter the UV sensitivities of the PCNA-WT or vector-transfected control cells, but did increase the sensitivity of the PCNA[KR] cells (Fig. 8E), suggesting that REV1 plays a complementary role in the DDT pathway disrupted by expression of mutant PCNA. In summary, these results indicate that mono-ubiquitination of PCNA promotes at least two distinct pathways: Polη-mediated TLS and a multi-mono-ubiquitination-dependent DDT mechanism (Fig. 8F).

## Discussion

PCNA plays crucial roles in various cellular processes, including DNA replication, DNA repair, and DDT pathways. PCNA functions as a homo-trimer and post-translational modifications of PCNA are important for controlling DDT pathways; however, the relevance of multiple modifications of a PCNA homo-trimer has not yet been elucidated. This study sheds light on the role of multiple mono-ubiquitinations of PCNA homo-trimers in the regulation of DDT pathways and unveils the regulatory mechanism of a minor DDT pathway that is masked by Polη-mediated TLS, the prominent DDT pathway, in human cells.

The results presented here show that multiple units in a PCNA homo-trimer are simultaneously ubiquitinated both *in vitro* and *in vivo* (Figs. 1 and 2). The results of an *in vitro* assay revealed that sequential mono-ubiquitinations of PCNA homo-trimers containing at least one ubiquitinated monomer occur more readily than the initial ubiquitination of a unit in unmodified trimers (Fig. 1). Since endogenous and exogenous PCNA were able to form hetero-complexes in human cells (Fig. 6A), we were able to reduce the levels of PCNA trimers capable of undergoing triple modifications by expressing PCNA[KR] at various levels. As expected, ectopic expression of PCNA[KR] reduced the level of multi-mono-ubiquitinated PCNA homo-trimers (Fig. 3B). Notably, cells expressing both endogenous PCNA and exogenous PCNA[KR] displayed slight but detectable increased sensitivities to UV irradiation and cisplatin (Fig. 4 A and B) and had DNA replication problems (Fig. 5A-D). DDT defects were evident in cells expressing HA-PCNA[KR] at levels sufficient to deplete endogenous PCNA homo-trimers but were mild in cells expressing lower levels of HA-PCNA[KR] (Fig. 6B). In addition, increasing the mono-ubiquitination of PCNA by transient expression of Polη or RAD18 did not correct the DDT problems caused by the expression of K164R-mutated PCNA (Fig. 7B), and slight but detectable multiple mono-ubiquitinations of a PCNA trimer were observed in PCNA[KR] cells overexpressing Polη (Fig. 7C). Since triple PCNA modifications were disturbed in cells expressing PCNA[KR], we speculated that the observed modifications occurred on two units. Therefore, it is likely that the multiple mono-ubiquitination-mediated DDT pathway requires modification of all three subunits of the PCNA trimer. These results suggest that depletion of multiple modification-susceptible PCNA homo-trimers, rather than an overall decrease in the level of mono-ubiquitination, is the cause of the disrupted DDT pathway in cells expressing PCNA[KR], although we cannot exclude the possibility that the presence of unmodifiable PCNA[KR] trimers also disturbs the DDT pathway.

Notably, the sensitivities of Polη-proficient and Polη-deficient cells to UV treatment were increased by PCNA[KR] expression (Figs. 4A and 8B), suggesting that the DDT pathway disrupted by PCNA[KR] expression is distinct from Polη-mediated TLS. UV-induced formation of Polη foci was detected in PCNA[KR] cells (Fig. 8C), which also suggests that multi-mono-ubiquitination is not required for Polη-mediated TLS. Polη is involved in producing somatic hypermutations that are dependent on PCNA ubiquitination [21]; however, in a study by Langerak *et al*. [22], TLS-directed mutation of sites around genetic lesions in activated B-cells was comparable in WT and PCNA[KR] heterozygous knock-in mice, suggesting that mono-ubiquitination of one unit of a PCNA homo-trimer is sufficient for activation of Polη, which is consistent with our observation that Polη is likely to act in PCNA[KR] cells. Furthermore, REV1 depletion increased the sensitivity of PCNA[KR] cells to UV irradiation (Fig. 8E), suggesting that the multiple mono-ubiquitination-mediated DDT and REV1-mediated TLS are likely to be independent. However we do not exclude a possibility that other TLS polymerases are involved in multiple mono-ubiquitination mediated DDT. Rather our results support a model that more than two TLS polymerases simultaneously bind to a PCNA ring and promote multiple two polymerase mechanisms [11, 12]. It has been reported previously that cells have mechanisms for controlling TLS independently of PCNA modifications [23, 24], indicating that these two processes are able to contribute to DDT independently, although a major contribution is achieved by their cooperation.

There are several reports that may support the importance of triple mono-ubiquitinations of PCNA trimers. The Ub moieties of mono-ubiquitinated PCNA are flexible [25, 26], suggesting that PCNA trimers containing three modifications are able to function normally; in support of this theory, PCNA complexes containing His-Ub modifications on all units have been observed in human cells [27]. Previous studies also demonstrated that Ub-fused PCNA mimics mono-ubiquitinated PCNA and corrects DDT defects of cells lacking mono-ubiquitinated PCNA [9, 10, 28, 29]. Furthermore, Ub-PCNA promotes not only Polη-dependent TLS, but also a Polη-independent DDT pathway in yeast and human cells [9, 10]. Overexpression of Ub-PCNA would be expected to mimic not only mono-ubiquitination, but also multi-mono-ubiquitinations of PCNA. Based on our observations and the results of previous studies, we propose that cells have Polη-mediated and Polη-independent DDT pathways, and that mono-ubiquitination of PCNA homo-trimers is sufficient to activate the former, whereas multi-mono-ubiquitinations are required to activate the latter (Fig. 8F).

The results presented here demonstrate that RAD18 preferentially catalyzes sequential multiple mono-ubiquitination of PCNA homo-trimers (Fig. 1), suggesting that RAD18 is involved in the regulation of a DDT pathway. However, we do not exclude the possibility that other Ub ligases with the potential to promote PCNA mono-ubiquitination, such as RNF8 [30], CRL4 (Cdt2) [31], and HLTF [32], also contribute to the DDT pathway. In addition to catalyzing multiple mono-ubiquitinations of PCNA trimers, RAD18 also catalyzes the simple mono-ubiquitination required for TLS [5] and can conjugate PCNA to K63-linked polyubiquitin chains produced by a concerted action with HLTF [33], which may promote the TS pathway [34]. This evidence suggests that multiple DDT pathways are regulated by RAD18-mediated ubiquitination.

Several proteins control the ubiquitination of PCNA, including Spartan/C1orf124/DVC1, NBS1, Polη, PTIP/Swift, CHK1-Claspin, and ELG1 [35]. Spartan/C1orf124/DVC1 interacts with PCNA and Ub, and controls Polη-mediated TLS and damage-induced mutagenesis [36–41]. NBS1 and Polη are thought to enhance the recruitment and/or activity of RAD18 [20, 42]. These accelerators have the potential to activate RAD18-regulated DDT mechanisms. In addition, de-ubiquitinating enzymes, particularly USP1, play crucial roles in the regulation of PCNA ubiquitination [43]. Spartan/C1orf124/DVC1 and ELG1 are thought to control the activity of USP1 [39, 44]. Our observations that the DDT pathway mediated by multi-mono-ubiquitinations of PCNA and Polη-mediated TLS could be discriminated by constitutive expression of K164R-mutated PCNA may help to elucidate the roles of regulators of PCNA modifications. Although poly-ubiquitination and SUMOylation modifications of PCNA were not detected in our system, we cannot exclude the possibility that different modifications of trimeric PCNA complexes occur simultaneously.

In summary, this paper sheds light on the relevance of co-modifications of multiple units of PCNA trimers. The information provided may represent a new twist on DDT regulatory mechanisms and may be relevant to other biological systems involving modified protein multimers.

## Supporting Information

S1 Fig Mono-ubiquitinations of PCNA-WT homo-trimers are out-competed by PCNA[KR] homo-trimers in a proportion-dependent manner. *In vitro* mono-ubiquitination assays of WT PCNA homo-trimers, WT homo-trimers mixed with PCNA[KR] homo-trimers at a ratio of 2:1 or 1:2, and PCNA hetero-trimers comprising one WT unit and two mutant units. The experiment was independent from that shown in Fig. 1C and D. (A) Immunoblot analysis using an anti-PCNA antibody. (B) The ratios of ubiquitinated to total PCNA at the indicated time points. Note that all reactions contained equal total amounts of PCNA trimers.
(TIF)

S2 Fig PCNA forms stable trimers *in vitro* and *in vivo*. Gel filtration analyses of PCNA. Recombinant PCNA (upper panel), recombinant PCNA exposed to the gel filtration buffer containing 1 M NaCl for 30 min (middle panel), and immunoprecipitants from HA-PCNA[KR] cells which were washed with a high salt buffer (lower panel) were subjected to a Superdex200 gel filtration column chromatography. The resulting fractions were analyzed by immunoblotting using an anti-PCNA antibody. The elution positions of catalase (Cat; 232 kDa), aldolase (Ald; 158 kDa), and bovine serum albumin (BSA; 67 kDa) are indicated.
(TIF)

S3 Fig Knockdown of endogenous PCNA but not exogenous PCNA by a specific siRNA. XP2SASV3/HA-PCNA-WT (WT) or /HA-PCNA[KR] (KR) cells were transfected with PCNA-specific siRNA (+) or a nontargeting control siRNA (-). Four days after transfection, whole cell lysates were prepared and analyzed by immunoblotting using anti-PCNA and anti-Lamin B antibodies.
(TIF)

S1 Methods (DOCX)
