# Supplementary figures and images for: Relevance of Simultaneous Mono-Ubiquitinations of Multiple Units of PCNA Homo-Trimers in DNA Damage Tolerance
Source: PLoS One. 2015 Feb 18;10(2):e0118775. doi: 10.1371/journal.pone.0118775 (PMC4332867; doi:10.1371/journal.pone.0118775)

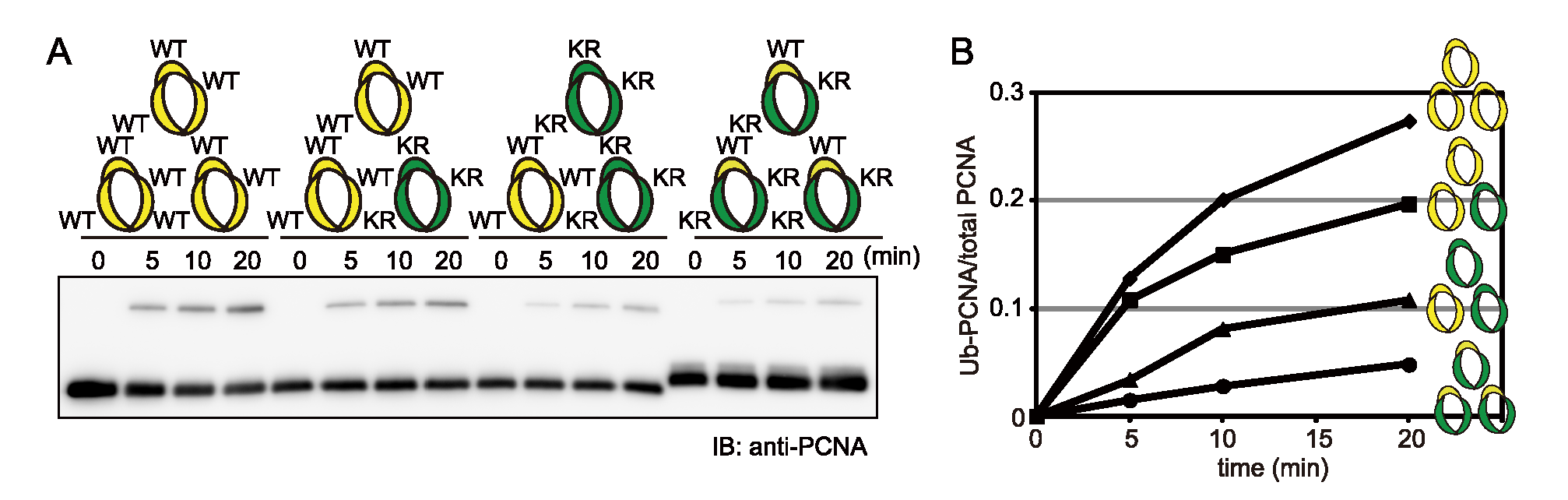

Supplement: S1 Fig — In vitro mono-ubiquitination assays of WT PCNA homo-trimers, WT homo-trimers mixed with PCNA[KR] homo-trimers at a ratio of 2:1 or 1:2, and PCNA hetero-trimers comprising one WT unit and two mutant units. The experiment was independent from that shown in Fig. 1C and D. (A) Immunoblot analysis using an anti-PCNA antibody. (B) The ratios of ubiquitinated to total PCNA at the indicated time points. Note that all reactions contained equal total amounts of PCNA trimers. (TIF) [file pone.0118775.s001.tif]

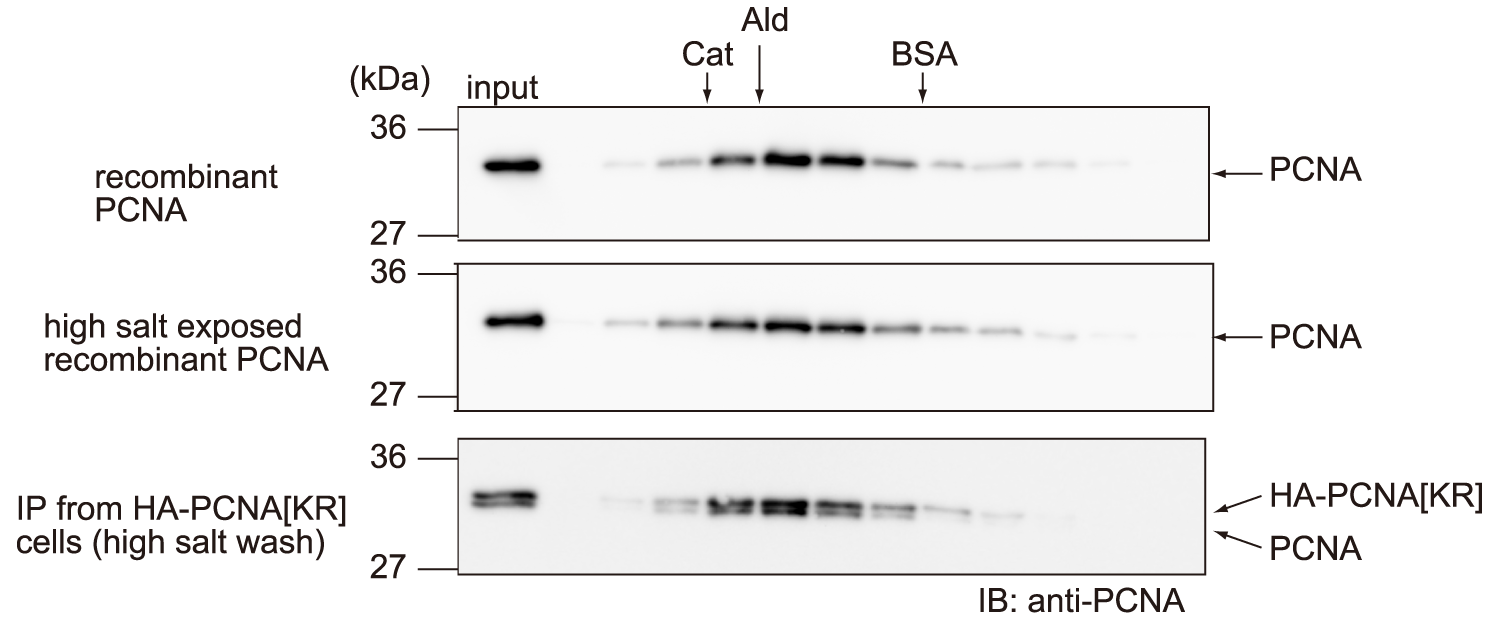

Supplement: S2 Fig — Gel filtration analyses of PCNA. Recombinant PCNA (upper panel), recombinant PCNA exposed to the gel filtration buffer containing 1 M NaCl for 30 min (middle panel), and immunoprecipitants from HA-PCNA[KR] cells which were washed with a high salt buffer (lower panel) were subjected to a Superdex200 gel filtration column chromatography. The resulting fractions were analyzed by immunoblotting using an anti-PCNA antibody. The elution positions of catalase (Cat; 232 kDa), aldolase (Ald; 158 kDa), and bovine serum albumin (BSA; 67 kDa) are indicated. (TIF) [file pone.0118775.s002.tif]

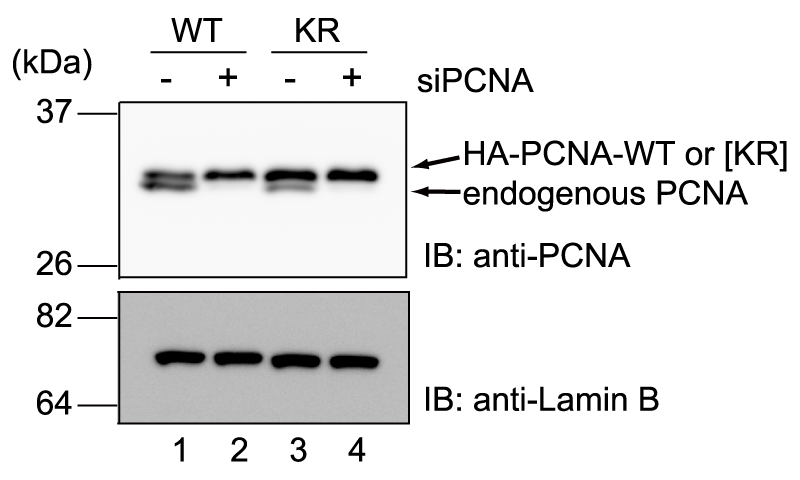

Supplement: S3 Fig — XP2SASV3/HA-PCNA-WT (WT) or /HA-PCNA[KR] (KR) cells were transfected with PCNA-specific siRNA (+) or a nontargeting control siRNA (-). Four days after transfection, whole cell lysates were prepared and analyzed by immunoblotting using anti-PCNA and anti-Lamin B antibodies. (TIF) [file pone.0118775.s003.tif]
