# Supplementary material for: Relevance of Simultaneous Mono-Ubiquitinations of Multiple Units of PCNA Homo-Trimers in DNA Damage Tolerance
Source: PLoS One. 2015 Feb 18;10(2):e0118775. doi: 10.1371/journal.pone.0118775 (PMC4332867; doi:10.1371/journal.pone.0118775)
Supplement: S1 Methods — (DOCX) [file pone.0118775.s004.docx]

**Supporting Methods**

**Gel filtration**

Purified recombinant PCNA and immunoprecipitants from HA-PCNA[KR] (clone #2) cells were loaded onto a Superdex200 PC 3.2/30 column (GE Healthcare) equilibrated with gel filtration buffer (50 mM Hepes-NaOH (pH 7.5), 0.1 M NaCl, 10% glycerol, 1 mM DTT, 0.1 mM EDTA, and 0.1% Triton X-100) at a rate of 10 μl min^-1^ using the SMART system (GE Healthcare). The eluted fractions were analyzed by immunoblotting. Preparation of the immunoprecipitates was performed as described in the main text, with the exceptions that a high salt buffer (50 mM Hepes-NaOH (pH 7.5), 1 M NaCl, 10% glycerol, 0.1% Triton X-100, 5 mM β-mercaptoethanol, and 0.25 mM phenylmethylsulfonyl fluoride) was used to wash the beads and the same high salt buffer containing 0.2 mg/ml HA peptide was used to elute the precipitants.
